# Synopsis of *Commelina* L. (Commelinaceae) in the state of Rio de Janeiro, reveals a new white-flowered species endemic to Brazil

**DOI:** 10.3897/phytokeys.78.11932

**Published:** 2017-04-05

**Authors:** Marco Octávio de Oliveira Pellegrini, Rafaela Campostrini Forzza

**Affiliations:** 1 Universidade de São Paulo, Departamento de Botânica, Rua do Matão 277, CEP 05508-900, São Paulo, SP, Brazil; 2 Jardim Botânico do Rio de Janeiro, Rua Pacheco Leão 915, CEP 22460-030, Rio de Janeiro, RJ, Brazil; 3 Current address: Smithsonian Institution, NMNH, Department of Botany, MRC 166, P.O. Box 37012, Washington D.C. 20013-7012, USA

**Keywords:** Atlantic Forest, Commelineae, *Commelinopsis*, Neotropical flora, *Phaeosphaerion*

## Abstract

A synopsis for the genus *Commelina* in the state of Rio de Janeiro, Brazil, is presented here, including a new species, ten new synonyms, five designated lectotypes, two designated epitypes and an excluded name. *Commelina huntii*, a new species, is remarkable due to the combination of rusty to rusty-brown hairs at the margin of its leaf-sheaths, connate spathes, white flowers with auriculate medial petal, ovaries with sparse black papillae and dehiscent fruits. Additionally, we provide an identification key, illustrations, and conservation status for the species of *Commelina* recorded in the state of Rio de Janeiro.

## Introduction

*Commelina* L. is the largest genus of Commelinaceae, comprising between ca. 170 species (Faden 1998) and 205–215 species (Govaerts and Faden 2009; The Plant List 2013, respectively). It is one of the six genera of Commelinaceae (out of 42) to have a cosmopolitan distribution (Faden 1998), and one of the most complicated taxonomically. *Commelina* is easily differentiated from the remaining genera in the tribe by its inflorescences which are subtended by spathaceous basal bracts and reduced to (1–)2 fasciculate cincinni, zygomorphic flowers, petals clawed, unequal and mostly blue (but sometimes white or lilac, rarely yellow, apricot or orange), three posterior staminodes with 6-lobed cruciform antherodes, three anterior stamens, and 2-locular or unequally 3-locular and 2-valved capsules (Faden 1998).

The state of Rio de Janeiro is entirely placed within the Atlantic Forest domain (IBGE 2012), being one of the four diversity centers of the family, and possessing 67 of the 96 accepted Commelinaceae species for Brazil (BFG 2015). With 1,109,546 ha of continuous forests, which represent 7% of what remains of the Atlantic Forest, Rio de Janeiro is also the state with the greatest amount of preserved forest remnants from this biome (Ribeiro et al. 2009). Nevertheless, the most recent published state flora is nearly two centuries old (*i.e.* Vellozo 1829) and no satisfactory taxonomic treatment for the Commelinaceae has been done since. As a first attempt to clarify the taxonomy of *Commelina* in the state of Rio de Janeiro, we describe a new species of *Commelina*, together with a synopsis for the genus in the state. This work includes an identification key, illustrations and an overview of some overlooked Brazilian *Commelina* names.

## Methods

The descriptions and phenology of the species were based on herbaria, spirit, fresh material, field data, and literature. All species were studied in the field and had their descriptions complemented with field notes, photographs, and cultivated specimens, gathered between the years of 2010–2016. Live specimens collected by the authors were kept in cultivation at the greenhouse of the Jardim Botânico do Rio de Janeiro, in order to better observe, photograph and analyze fresh flowers, fruits and seeds, as well as other phenological data. Specimens from the following herbaria were also analyzed: ALCB, BHCB, BHZB, BM, BOTU, CEPEC, CESJ, CGE, CNMT, CVRD, ESA, FCAB, FLOR, FURB, GUA, HAMAB, HAS, HB, HBR, HRB, HSTM, HUEFS, HURB, IAC, ICN, JOI, K, MBM, MBML, PMSP, R, RB, RFA, RFFP, SP, SPF, UEC, UPCB, and US (herbaria acronyms according to Thiers, continuously updated). While specimens of the following herbaria were analyzed using high-resolution images available on-line: B, BRIT, C, CAL, F, INPA, L, MG, MO, MY, NY, P, U, and WAG. The classification of the vegetation patterns follows IBGE (2012). The indumenta and shapes terminology follows Radford et al. (1974); the inflorescence terminology and morphology follows Weberling (1965, 1989) and Panigo et al. (2011); the fruit terminology follows Spjut (1994) and Joseph & Nampy (2012); and seeds terminology follows Faden (1991) and Joseph & Nampy (2012). The conservation statuses were proposed following the recommendations of *IUCN* *Red List Categories and Criteria, Version 3.1* (IUCN 2001). GeoCAT (Bachman et al., 2011) was used for calculating the Extent of Occurrence (EOO) and the Area of Occurrence (AOO). The typification of Vellozo’s names for *Commelina* followed the same methodology used by Pellegrini (2015), Pellegrini et al. (2015) and Pellegrini & Carvalho (2016).

## Results

### Key to the species of *Commelina* in Rio de Janeiro state, Brazil

**Table:** 

1	Inflorescences predominantly axillary and leaf-opposed, long pedunculate (peduncle the same length or longer than ½ length of the spathe); medial petal clawed	***Commelina diffusa* Burm.f.**
–	Inflorescences terminal or apparently so, short-pedunculate to sessile (peduncle shorter than ½ length of the spathe); medial petal sessile	**2**
2	Spathe base free, *in vivo* much lighter than the leaves; capsules indehiscent, not constricted between the seeds, crustaceous, pearly-white to silvery; all seeds adhered to the capsule wall and septa, forming a dispersal unit	**3**
–	Spathe base connate, *in vivo* the same color as the leaves; capsules dehiscent, constricted between the seeds, green to light brown; dorsal seeds adhered to the capsule wall, ventral seeds free from each other, dispersed separately	**4**
3	Leaf-sheaths hirsute throughout, hairs rusty to rusty-brown, blades lanceolate to elliptic-lanceolate, hispid on both sides, hairs hyaline, sparsely hirsute along the midvein and near base, hairs rusty to rusty-brown, base cuneate, apex acute	**Commelina rufipes Seub. var. rufipes**
–	Leaf-sheaths glabrous, margin glabrous to setose, hairs rusty to rusty brown, blades ovate-elliptic to ovate, glabrous, base round to obtuse, apex acuminate to caudate	**Commelina rufipes var. glabrate (D.R.Hunt) Faden & D.R.Hunt**
4	Leaf-sheaths with auriculate margin; upper cincinnus aborted, included; medial petal hyaline; capsules 3-seeded, dorsal locule commonly verrucous, rarely smooth; testa smooth	***Commelina erecta* L.**
–	Leaf-sheaths with patent and erect margin; upper cincinnus present, exerted; medial petal slightly paler to concolorous with the paired petals; capsules 5-seeded, dorsal locule smooth; testa ornate	**5**
5	Leaves subpetiolate; spathe transversally rhomboid; cleistogamous subterraneous flowers present, medial petal trullate, ovary minutely pilose, stigma capitate; capsules ellipsoid; seeds black, testa shallowly reticulate	***Commelina benghalensis* L.**
–	Leaves sessile; spathe depressed ovate to subcordate; cleistogamous subterraneous flowers absent, medial petal obovate to oblong obovate, ovary glabrous, stigma trilobate; capsules obovoid; seeds dark-brown, testa shallowly foveolate, foveolate or rugose foveolate	**6**
6	Leaf-sheath margin densely bearded with rusty to rusty brown hairs; petals white, paired petals broadly rhomboid to rhomboid-reniform, medial petal cucullate, biauriculate; ovary and capsules with black papillae, 1–2 capsules per spathe; seeds with peach-colored farina, dorsal locule seed testa shallowly foveolate	***Commelina huntii* M.Pell.**
–	Leaf-sheath margin with light to dark brown to atro-vinaceous hairs; petals blue to light-blue to lilac to pale lilac, paired petals broadly ovate to broadly ovate-reniform, medial petal involute, entire; ovary and capsules smooth, 5–7 capsules per spathe; seeds with white-farinose, dorsal locule seed testa rugose-foveolate	***Commelina obliqua* Vahl**

#### 1. Commelina benghalensis

Taxon classificationPlantaeCommelinalesCommelinaceae

L., Sp. Pl. 1: 41. 1753 nom. cons.

Fig. 1A

##### Neotype

(conserved and designated by Faden 1992). INDIA. Habitat in Benghala, s.dat., s.leg., Herb. Linn. 65.16 (LINN!).

##### Selected specimens seen.

BRAZIL. Rio de Janeiro: Angra dos Reis, Ilha Grande, matas da praia de Abraão, 18 Apr 1987, L.C. Giordano 277 (RB). Armação de Búzios, 19 Aug 1998, D. Fernandes 19 (R). Cabo Frio, 22 Oct 2013, H.F. Uller s.n. (RB 612224). Campos dos Goytacazes, Morro do Itaoca, Pedra Negra ponto 1, 13 Oct 2009, L.P. Mauad & I.O.R. Areias 20 (RB). Casimiro de Abreu, Praião, Avenida Oceânica, 24 Jun 2012, A.J. Castelo 39 (RB). Iguaba Grande, Km 94, 1981, H. Barreto s.n. (RB 275353). Macaé, Córrego de Ouro, Fazenda Vitória, Morro do Oratório, 2 May 1971, J.P.P. Carauta 1364 (RB, U n.v.). Niterói, Itaipuaçu, próximo ao Canal da Costa, 18 Sep 2004, T.T. Carrijo 143 (RB). Resende, margem da rodovia Dutra, Km 302 sentido RJ, ao lado da Light, próximo ao Rio Paraíba do Sul, 9 Jun 2012, M.O.O. Pellegrini et al. 233 (RB). Rio de Janeiro, Morro do Rangel, Recreio dos Bandeirantes, 31 May 1973, D. Sucre 10005 (RB, US). São José de Ubá, 14 May 2014, T.M. Scarponi s.n. (RB 612228). Saquarema, Tingui em Sampaio Correia, 14 Apr 1995, J.A. Lira Neto 56 (RB). Silva Jardim, Próximo a sede da REBIO, 29 Oct 1997, J.A. Lira Neto 719 (RB).

##### Distribution and habitat.

Tropical and subtropical regions of the world. In the state of Rio de Janeiro it is especially common in disturbed areas of drier regions inland or near the coast, being common in *restingas* (*i.e.* sandbank vegetation), and as a weed in agricultural fields (Fig. 2).

##### Phenology.

Throughout the year, but especially in the rainy season.

##### Conservation status.

It is a weed of worldwide distribution being very common in sunny disturbed areas and in agricultural fields. Following the IUCN recommendations (IUCN 2001), it should be considered as Least Concern (LC) in the state of Rio de Janeiro and worldwide.

##### Morphological and ecological notes.

The underground cleistogamous flowers and fruits seem to be produced only in areas where the soil is soft. The flower morphology differs from the aerial chasmogamous in pigmentation (being paler), while the fruits are sub-globose (due to larger seeds).

#### 2. Commelina diffusa

Taxon classificationPlantaeCommelinalesCommelinaceae

Burm.f., Fl. Indica 18: pl. 7, f. 2. 1768.

Fig. 1B

Commelina communis Vell., Fl. Flumin.: 30, 1829, nom. illeg. non C. communis L., Sp. Pl. 1: 40, 1753. Lectotype (designated here). [illustration] Original parchment plate of *Flora fluminensis* in the Manuscript Section of the Biblioteca Nacional do Rio de Janeiro [mss1095062_079] and later published in Vellozo, Fl. flumin. Icon. 1: t. 75. 1831. **Syn. nov.**

##### Holotype.

INDIA. Coromandel, s.dat., D. Outgaerden s.n. (G barcode G00360106!).

**Figure 1.**
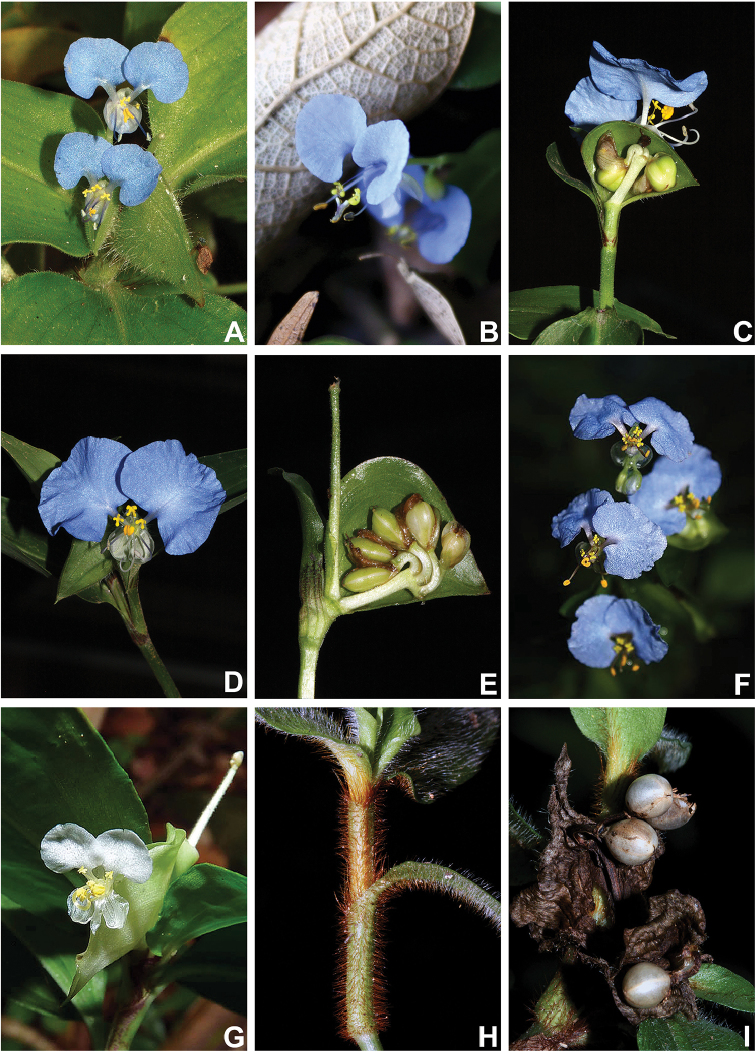
*Commelina* in the state of Rio de Janeiro. **A** *C. benghalensis* L. **B** *C. diffusa* Burm.f. **C–D** *C. erecta* L.: **C** detail of the inflorescence showing aborted upper cincinnus **D** flower **E–F** *C. obliqua* Vahl: **E** detail of the inflorescence showing the two developed cincinni **F** flower **G** C. rufipes var. glabrata (D.R.Hunt) Faden & D.R.Hunt **H–I** C. rufipes var. rufipes Seub.: **H** detail of the leaf-sheaths, showing the hirsute rusty hairs **I** mature fruits. G by Flora Virtual Estación Biológica El Verde group, remaining field photos by M.O.O. Pellegrini.

**Figure 2.**
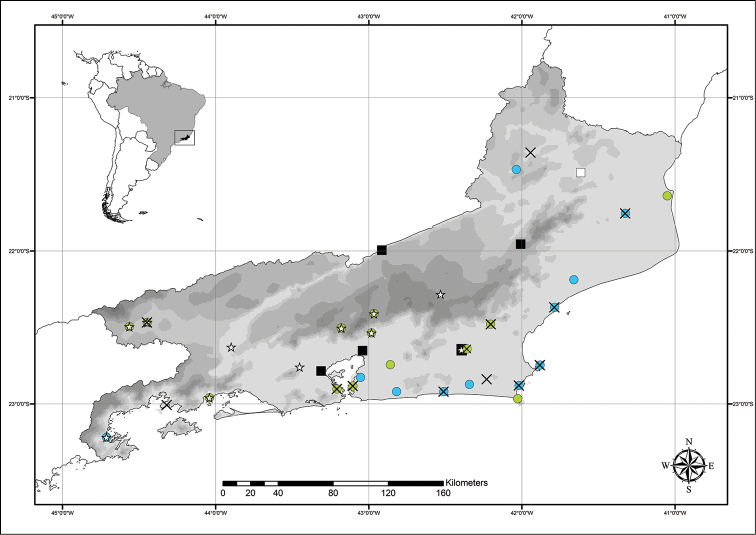
Distribution map of *Commelina* in the state of Rio de Janeiro. **Crosses** *C. benghalensis* **green dots** C. diffusa var. diffusa; **blue dots** *C. erecta*; **stars** *C. obliqua*; **empty square** C. rufipes var. glabrata; **full squares** C. rufipes var. rufipes.

**Selected specimens seen.** BRAZIL. Rio de Janeiro: Arraial do Cabo, 24 Jul 1953, F. Segadas Vianna 1157 (R, US). Casimiro de Abreu, Barra de São João, 27 May 1953, F. Segadas Vianna 347 (R, US). Guapimirim, trilha das andorinhas, 20 Dec 1995, L.A. Lira Neto 161 (RB). Itaboraí, entre os rios Caceribu e Macacu, 10 Aug 2007, A. Rodarte 4Cf (RB). Itatiaia, Parque Nacional do Itatiaia, Hotel Repouso, 14 Dec 1997, J.M.A. Braga 4539 (RB). Mangaratiba, Reserva Ecológica Rio das Pedras, trilha para o mirante, 12 Jul 1997, J.A. Lira Neto 603 (RB). Niterói, Jurujuba, 16 Jan 1959, A. Castellanos 22336 (R). Petrópolis, Serra da Estrela, meio da Serra, antigo leito da estrada de ferro, próximo ao Poço do Cipó, 9 Mar 1978, P.P. Jouvin 121 (RB). Resende, margem da rodovia Dutra, Km 302 sentido RJ, ao lado da Light, próximo ao Rio Paraíba do Sul, 9 Jun 2012, M.O.O. Pellegrini et al. 232 (RB). Rio de Janeiro, Urca, 10 Jan2012, M.O.O. Pellegrini et al. 182 (RB). São João da Barra, 30 May 1953, F. Segadas Vianna 428 (R, US). Silva Jardim, próximo à sede da REBIO, 29 Oct 1997, J.A. Lira Neto 714 (RB). Teresópolis, Laje, estrada para Campo Limpo, Granja Mafra, 28 May 1977, L.D.A. Freire de Carvalho 600 (RB).

##### Distribution and habitat.

Tropical and subtropical regions of the world, being very common in shady disturbed areas such as road sides, gardens and forest margins, and in agricultural fields. It is also found growing on the edge of wet paddy fields, ponds, ditches and stream sides (Fig. 2).

##### Phenology.

Throughout the year, but especially in the rainy season.

##### Conservation status.

Following the IUCN recommendations (IUCN 2001), it should be considered as Least Concern (LC) in the state of Rio de Janeiro and worldwide.

##### Morphological notes.

The specimens from the state of Rio de Janeiro usually show a staminode malformation, *i.e.* the central antherode is lacking in most of the herbaria and living specimens examined.

##### Nomenclatural and taxonomical notes.

*Commelina communis* Vell. is a later homonym of *C. communis* L. (the genus’ type species), thus, rendering it illegitimate. Vellozo’s description (1929) is little informative, especially for *Commelina*, lacking all the characters evidenced by Faden (2008) as important to delimitate species in the genus. Despite this, the spathe and flower details (Vellozo 1831: t. 75), along with the leaf shape and stem diagnosis (Vellozo 1929), make it possible to associate *C. communis* Vell. to *C. diffusa*, rather than to *C. deficiens* Herb. (= *C. erecta*), as pointed out in the *Index Methodicus* of *Flora fluminensis* (Vellozo 1831, v. 1).

#### 3. Commelina erecta

Taxon classificationPlantaeCommelinalesCommelinaceae

L., Sp. Pl. 1: 41. 1753.

Fig. 1C–D

Commelina erecta var. angustifolia (Michx.) Fern., Rhodora 42(503): 439.1940. **Syn. nov.**
Commelina virginica var. angustifolia (Michx.) C.B.Clarke, in De Candolle ALPP & De Candolle ACP Monogr. Phan. 3: 183. 1881. **Syn. nov.**
Commelina angustifolia Michx., Fl. Bor.-Amer. 1: 24. 1803. Holotype. USA. Sabulosis in Carolinae, s.dat., A. Michaux 100 (P barcode P00680427!). **Syn. nov.**
Eudipetala deficiens (Herb.) Raf., Fl. Tellur. 3: 68. 1837. **Syn. nov.**
Commelina deficiens Herb., Bot. Mag. 53: t. 2644. 1826. Lectotype (designated here). [illustration] Original parchment plate of “Curtis’s Botanical Magazine” at the Library of the Royal Horticultural Society, published in Hooker, Curtis’s Bot. Mag. 53: t. 2644. 1826. **Syn. nov.**
Commelina erecta f. villosa (C.B.Clarke) Stand. & Steyerm., Publ. Field Mus. Nat. Hist., Bot. Ser. 23(2): 33. 1944. **Syn. nov.**
Commelina villosa (C.B.Clarke) Chodat & Hassl., Bull. Herb. Boissier, sér. 2, 1: 438. 1901. **Syn. nov.**
Commelina virginica var. villosa C.B.Clarke, Monogr. Phan. 3: 183. 1881. Lectotype (designated here). BRAZIL. Rio Grande do Sul: “provincia de Rio Grande do Sul”, 1816–1821, A. St.-Hilaire 2598 (P barcode P01742038!; isolectotype: P barcode P01742041!). **Syn. nov.**

##### Lectotype

(designated by Clarke 1881). “*Commelina erecta, ampliore subcaeruleo flore*” in Dillenius, Hort. Eltham. 1: 91, t. 77, f. 88. 1732.

##### Selected specimens seen.

BRAZIL. Rio de Janeiro: Araruama, 20 Apr 2008, A.C.S. Cavalcanti 139 (SPF). Armação de Búzios, loteamento de João Fernandes, quadra A, rua I, lote 10, 27 Jul 2013, M. Furtado 28 (RB). Arraial do Cabo, Praia do Pontal, 31 Jul 1953, F. Segadas Vianna s.n. (US barcode US 2283943.2455262). Cabo Frio, Peró, Sítio Guriri, 21 Jul 2003, G.S.Z. Rezende 191 (RB). Campos dos Goytacazes, Feb 1918, A.J. Sampaio 2813 (R). Carapebus, 23 Mar 1996, V. Esteves 947 (R). Macaé, Parque Nacional da Restinga de Jurubatiba, margem da estrada principal, entre a praia e as moitas, próximo a Lagoa Cabiúnas, 23 Jun 2013, L.S.B. Calazans 219 (RB). Mangaratiba, Ilha da Marambaia, Praia Grande, 15 Jan 1986, E.M. Occhioni 484 (RB). Maricá, 16 Feb 2005, A.T.A. Rodarte 195 (RB). Niterói, Parque Estadual da Serra da Tiririca, Pedra de Itacoatiara, 16 Feb 2000, M.C.F. Santos 496 (RB, RFFP). Paraty, Parati Mirim, Fazenda Parati-Mirim, propriedade da Flumitur, s.dat., C. Almeida 1931 (RB). Rio de Janeiro, Urca, 10 Jan 2012, M.O.O. Pellegrini et al. 181 (RB). Santo Antônio de Pádua, Monte Alegre, Mar 1927, J. Vidal s.n. (R 205994). São Gonçalo, Paraíso, Faculdade Formação de Professores da Universidade do Estado do Rio de Janeiro, 20 Oct 2006, N. Coqueiro 297 (RB, RFFP). São João da Barra, Restinga de Iquipari, 11 Dec 2002, M.C. Gaglione 8 (RB). Saquarema, 21 Feb 1996, A.Q. Lobão 76 (RB).

##### Distribution and habitat.

Tropical and subtropical regions of the world, being common in disturbed areas of drier regions inland or near the coast, commonly found in *restingas* or in urban areas (Fig. 2).

##### Phenology.

Throughout the year, but especially in the rainy season.

##### Conservation status.

Following the IUCN recommendations (IUCN 2001), it should be considered as Least Concern (LC) in the state of the Rio de Janeiro and worldwide.

##### Nomenclatural and taxonomical notes.

Clarke (1881), in his revision of Commelinaceae, erroneously considered *C. erecta* as a synonym of *C. virginica* L., a species endemic to the USA (Faden 2000). Thus, some names currently placed under the synonymy of *C. erecta* were originally described under *C. virginica*, or transferred to it at some point. According to Faden (1993, 2000), *Commelina erecta* can be differentiated by its leaf-sheaths with auriculate margins, medial petal linear and hyaline, and all locules 1-seeded (*vs.* leaf-sheaths not auriculate, medial petal blue and conspicuous, and dorsal locule 1-seeded and ventral locules 2-seeded, in *C. virginica*).

There seems to be some doubt regarding *C. deficiens* Herb. synonymy. According to Tropicos.org (2015), this species is considered a synonym of *C. erecta*. Nevertheless, eMonocot.org (2010) and The Plant List (2013) treat *C. deficiens* under the synonymy of *C. virginica*. As abovementioned, there is an historical confusion regarding *C. erecta* and *C. virginica*. If we exclusively take into account that *C. deficiens* was described by Herbert (1826) from the surrounding areas of Rio de Janeiro, it is impossible for *C. deficiens* to be conspecific to *C. virginica*. Added to that, the watercolour presented by the author perfectly illustrates the habit, flower morphology and the inflorescence characteristic of *C. erecta* (with the aborted upper cincinnus). Thus, there is little doubt that *C. deficiens* is a synonym of the latter. According to Stafleu and Cowan (1979), Herbert’s type specimens were deposited at K, but no specimen corresponding to *C. deficiens* was found. Thus, in accordance to the *Code* (McNeill et al. 2012, Art. 9.12), in the absence of herbarium specimens, we designate this illustration as the lectotype for *C. deficiens*.

*Commelina villosa* (C.B.Clarke) Chodat & Hassl. has long been a name of dubious application. Clarke (1881) had already noticed that its morphological concept overlapped with the one of the highly variable *C. erecta*, and that the difference between them relied solely on the plant’s indumenta. The observation of a great number of natural populations and specimens kept in greenhouses showed that most of the morphological variation known for *C. erecta* has an environmental background. Large flowered specimens developed into small flowered specimens after being transplanted from sunny to shady areas. The same thing happened to narrow-leafed and erect plants (which would represent C. erecta var. angustifolia), developing into creeping and small to wide-leafed plants. The indumenta also varied when specimens were transplanted from the field to the greenhouse. Regarding growth form and position of the stem of *C. erecta*, the plants can vary from creeping to sub-scandent (*i.e.* stems leaning generally on bushes or any other kind of support) to partially or completely erect. The only morphological characters, constant in all areas and environmental conditions were: the auriculate leaf-sheath margins; terminal to apparently terminal inflorescences (1–3 per stem), broadly sagittate to subcordate spathes with connate margin, aborted upper cincinnus (generally completely absent, but sometimes only vestigial); hyaline, linear and involute medial petal (almost invisible at blind sight); capsules with 1-seeded locules; and smooth seed testa.

After analyzing the original descriptions and the type specimens, it became clear that *C. villosa* and C. erecta var. angustifolia are conspecific to *C. erecta*. Thus, no varieties or subspecies are accepted in Brazil for *C. erecta*.

#### 4. Commelina huntii

Taxon classificationPlantaeCommelinalesCommelinaceae

M.Pell. sp. nov.

urn:lsid:ipni.org:names:60474145-2

Figs 3 , 4 , 5

##### Diagnosis.

*Commelina rufipes* Seub. *affinis, sed ab ea spathis depressum-ovatum vel sub-cordato, basi adnata, petalo inferioris auriculata, ovarium sparse nigro-papillose, capsulae ellipsoide dehicens, parda, seminibus libera differt.*

**Figure 3.**
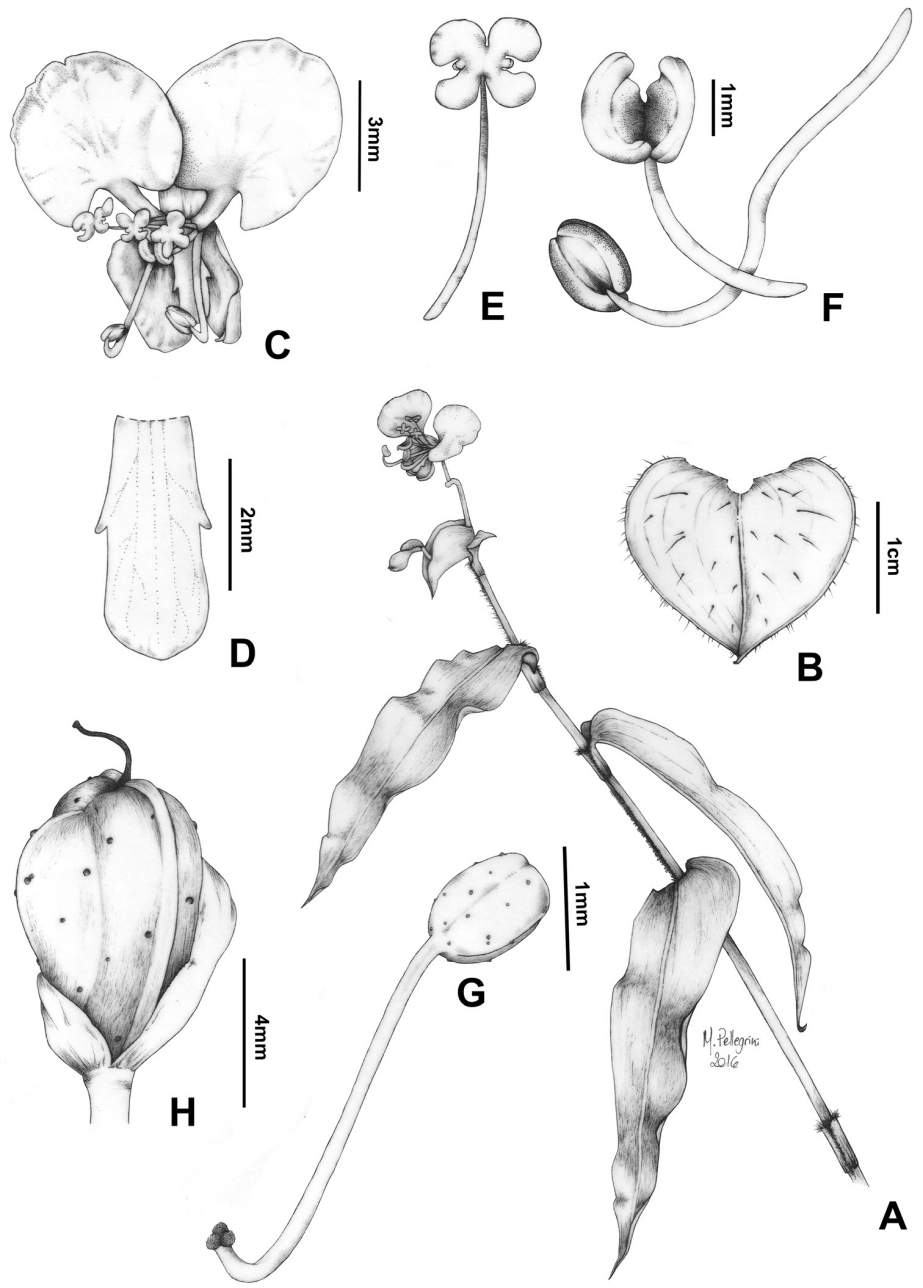
*Commelina huntii* M.Pell. **A** habit **B** open spathe, showing eventual rusty cilia and villous margin **C** male flower **D** medial petal, showing auricles **E** staminode **F** lateral stamen and medial stamen **G** gynoecium, showing papillate ovary and trilobate stigma **H** capsule, showing the black papillae. Line drawings by M.O.O. Pellegrini, based on the holotype.

**Figure 4.**
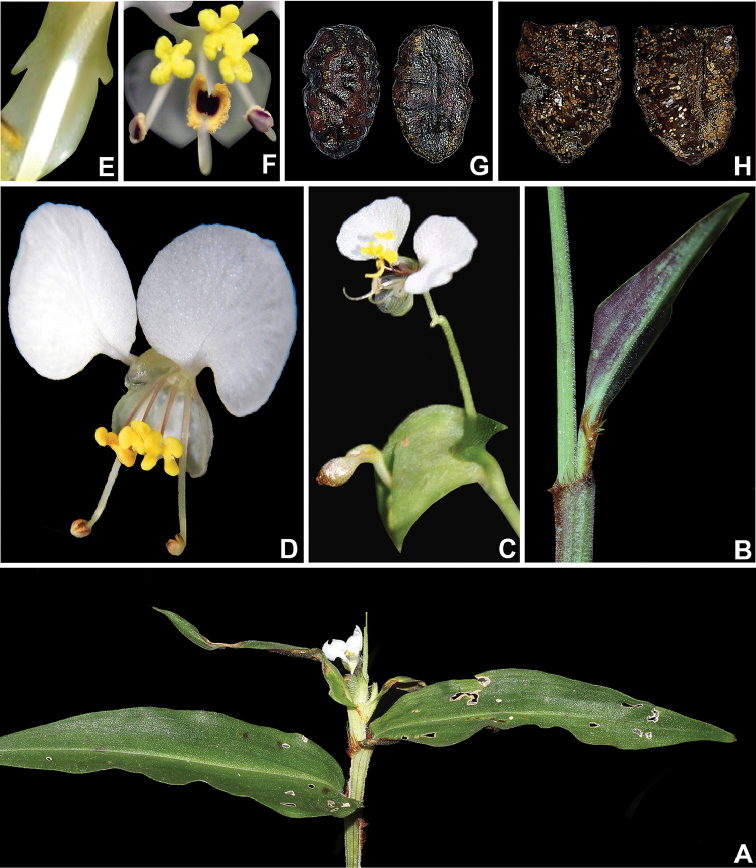
*Commelina huntii* M.Pell. **A** apex of the stem, showing terminal inflorescence **B** detail of the densely setose leaf sheath margins, with rusty hairs **C** detail of the inflorescence, showing the fused spathe and developed upper and lower cincinni **D** detail of a male flower **E** detail of the medial petal, showing the two auricles **F** detail of the androecium **G** dorsal and ventral view of the seed of the dorsal locule **H** dorsal and ventral view of one of the seeds of the ventral locules. **A** by L.S.B. Calazans, **B, C, E, G, H** by M.O.O. Pellegrini, **D** by M.S. Wängler and **F** by R.S. Couto.

##### Holotype.

BRAZIL. Rio de Janeiro: Itatiaia, Parque Nacional do Itatiaia, subida para o brejo da Lapa, beira de estrada, fl., fr., 24 January 2012, M.O.O. Pellegrini & L.S. Sylvestre 191 (RB!; isotypes: SPF!, US!).

##### Description.

*Herbs* 15–35 cm tall, perennial, terrestrial. *Roots* thin, fibrous, cream colored to light yellow, glabrous or minutely pubescent with absorbent hairs. *Stems* decumbent, apex ascending, becoming trailing or straggling, rooting only near the base; internodes 2.2–11.1 cm long, green, minutely velutine to minutely pilose, with line of uniseriate hairs opposite to the leaves, hairs hyaline. *Leaves* distichously-alternate, distributed along the stem, sessile; sheaths 1.4–2.6 cm long, pilose, hairs hyaline, margins densely setose, with a line of setose hairs opposite to the leaves, hairs rusty to rusty-brown; blades 3.3–9.1 × (0.9–)1.6–2.3(–3.3) cm, chartaceous, adaxially dark-green to green, abaxially light-green to light-green tinted vinaceous to completely vinaceous, drying olive-green on both sides, lanceolate to ovate lanceolate, rarely ovate, adaxially scabrous, abaxially minutely villous, pilose along the midvein, hairs hyaline, base obtuse, rarely cuneate, margins green, scabrous, apex acuminate; midvein conspicuous, impressed adaxially, prominently obtuse abaxially, secondary veins (2–)4–6 pairs, adaxially conspicuous, abaxially inconspicuous. *Inflorescences* 1–4, terminal or apparently so, peduncles 1.3–5.5 mm, rarely inconspicuous, puberulous with hook hairs throughout, hairs hyaline; spathes 0.7–2 × 1.4–3.2 cm, depressed ovate to subcordate, usually slightly falcate, base connate for 3–6 mm, cordate to truncate, margin green to vinaceous, minutely pilose along the edge, hairs hyaline, sometimes also ciliate, cilia rusty to rusty-brown, apex acute, internally light-green, glabrous, veins inconspicuous, externally green, minutely villous with eventual cilia, hairs hyaline, cilia rusty to rusty-brown, veins inconspicuous, becoming conspicuous when dry; upper cincinnus 2–5-flowered, flowers male, very rarely bisexual, peduncle (0.7–)1.7–2.4 cm long, exserted from the spathe, commonly arcuate at post-anthesis, sparsely to densely puberulous with hook hairs, sometimes of 2 heights, hairs hyaline; lower cincinnus 2–4-flowered, flowers mainly bisexual, sometimes male, peduncle 0.5–1 cm, glabrous or sparsely puberulous with minute hook hairs. *Flowers* bisexual or male, zygomorphic, 6.5–9 mm diam.; pedicel 1–4 mm long, light green, glabrous, reflexed and slightly elongate in fruit; sepals hyaline white to hyaline light-green, glabrous, persistent in fruit, upper sepal 3,4–4,2 × 1,1–1,4 mm, elliptic, cucullate, round apex, lower sepals 4.1–5.3 × 2.2–2.6 mm, obovate, cucullate, connate, round apex; paired petals 6.2–6.9 × 4.9–5.4 mm, clawed, limb broadly rhomboid to rhomboid-reniform, 4.7–5.3 × 4.9–5.4 mm, white, apex rounded, base cordate, claw 1.6–2 mm, white to tinted vinaceous, medial petal 3.1–4 × 1–1.4 mm, sessile, obovate to oblong-obovate, with 2 auricles near the middle, cucullate, concolorous with or slightly paler than the paired petals; staminodes 3, subequal, filaments 3–3.6 mm long, tinted vinaceous, antherodes 6-lobed, 1–1.2 × 1.2–1.4 mm, yellow with tiny light-yellow pollen sacs; lateral stamen filaments gently sigmoid, geniculate distal to the middle, 5.6–6.6 mm long, white, anthers elliptic to oblong-elliptic, 1.2–1.4 × 0.9–1.2 mm, yellowish-orange to cream-orange with margins tinted purple, pollen yellowish-orange to cream-orange; medial stamen filament straight or arcuate-decurved, decurved at the apex, 2.2–2.8 mm long, white to tinted vinaceous, anther 1.5–2.2 × 1–1.8 mm, broadly elliptic to broadly oblong-elliptic, strongly curved, held near the antherodes, yellow-orange to cream-orange, connective purple to dark-purple, pollen yellowish-orange to cream-orange; ovary oblong-ellipsoid, ca. 1–1.3 × 0.6–0.7 mm, 5-ovulate, glabrous, sparsely papillose, papillae black, style exceeding or equaling the stamens, sigmoid, strongly recurved apically, 8–11.3 mm, white, stigma trilobate, white. *Capsules* 1–2 per spathe, 5.5–8.1 × 3.8–5 mm, obovoid, constricted between the seeds, brown to light brown, glabrous, sparsely papillose, papillae black, 3-locular, 2-valved, dorsal locule 1-seeded, indehiscent, ventral locules 2-seeded. *Seeds* slightly dimorphic, dark brown with orange-brown verrucae, farinose, farina peach-colored; dorsal locule seed strongly adhered to the capsule wall, ellipsoid, strongly dorsiventrally compressed, ventrally flattened, not cleft towards the embryotega, 3.4–4.2 × 2.8–3.3 mm, testa shallowly foveolate, embryotega semilateral, relatively inconspicuous, without a prominent apicule, hilum linear, ½ the length of the seed; ventral locule seeds free from the capsule wall, ellipsoid, truncate at one end, ventrally flattened, not cleft towards the embryotega, 2.7–3.4 × 2–2.4 mm, testa foveolate, embryotega semilateral, relatively inconspicuous, without a prominent apicule, hilum linear, ½ the length of the seed.

##### Specimens seen

**(paratypes).** BRAZIL. Minas Gerais: Araponga, Parque Estadual Serra do Brigadeiro, Fazenda Neblina, 17 February 2005, L.S. Leoni et al. 6112 (RB, UEC); Camanducaia, Monte Verde, Serra da Mantiqueira, 11 December 2001, L.D. Meireles & R. Belinello 775 (HURB, UEC); Delfim Moreira, Fazenda da Onça, trilha de subida para o Pico do Carrasco, 17 March 2011, L.L. Giacomin et al. 1429 (BHCB, RB); Lima Duarte, Parque Estadual do Ibitipoca, Conceição do Ibitipoca, gruta da Cruz, 30 November 2004, E.V.S. Medeiros et al. 364 (RB); loc. cit., gruta do Cruzeiro, 20 January 2005, L.G. Temponi et al. 407 (RB, UEC); loc. cit., gruta do Pião, 18 January 2005, R.C. Forzza et al. 3926 (RB, UEC); loc. cit., gruta do Cruzeiro, 26 January 2010, J.C. Lopes et al. 76 (RB, SPF); Poços de Caldas, Fazenda Campo da Cachoeira, área destinada para a instalação do Jardim Botânico de Poços de Caldas, 12 December 2001, C.N. Fraga & F.M. Fernandez 864 (RB); loc. cit., 12 December 2001, F.M. Fernandez 151 (BHZB, RB). Rio de Janeiro: Nova Friburgo, Morro da Caledônia, 8 June 1977, G. Martinelli 2469 (BA, RB); loc. cit., Reserva Macaé de Cima, cerca de 900 m do Hotel São João, 19 January 1999, L. Anderson et al. 99/15 (UEC); loc. cit., Reserva Macaé de Cima, cerca de 900 m do Hotel São João, 19 January 1999, L. Anderson et al. 99/20 (UEC); loc. cit., Parque Estadual dos Três Picos, Vale dos Deuses, 28 January 2015, M.S. Wängler et al. 1565 (RB); Resende, Parque Nacional do Itatiaia, estrada BR-485, próximo ao km 02, 22 February 2014, L.S.B. Calazans et al. 242 (RB). São Paulo: Itararé, divisa entre as Fazendas Santa Andreia e Prieto, 14 May 1989, C.A.M. Scaramuzza & V.C. Souza 259 (ESA); Ribeirão Grande, Parque Estadual Intervales, 15 April 2003, R.A.G. Viani et al. 79 (ESA).

##### Etymology.

This species is named after the British botanist Dr. David R. Hunt, in honor of his extensive contribution to Commelinaceae systematics worldwide, especially for his contributions to Tradescantieae and the “*Phaeosphaerion* group” of *Commelina*.

##### Distribution and habitat.

**Figure 5.**
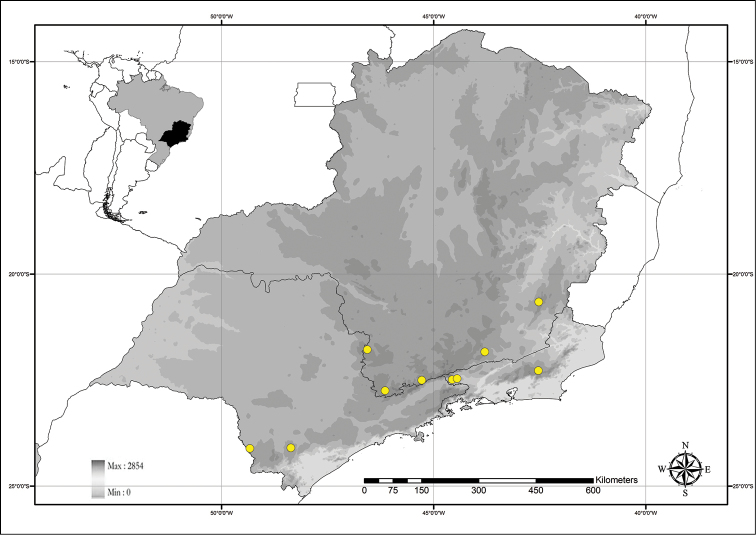
Distribution map of *Commelina huntii* M.Pell.

*Commelina huntii* was collected in moist and shaded nebular forests, generally near water bodies, in the states of Minas Gerais, Rio de Janeiro, and São Paulo, in elevations from 800 to 1,700 m above sea level (Fig. 5). In very rare cases it can also be found in open sometimes disturbed areas.

##### Phenology.

It was found in bloom from November to June and in fruit from December to March, rarely in June.

##### Conservation status.

Despite the wide EOO (112,904.528 km^2^), the AOO (40.000 km^2^) is considerably small, since all known populations are significantly small and fragmented. Following the IUCN recommendations (IUCN 2001), *C. huntii* should be considered as Endangered [EN, B2b(ii, iii)c(iv)+C2a(i)] in its overall distribution.

##### Affinities.

*Commelina huntii* can be recognized by its white flowers with auriculate medial petal and sparsely papillose ovary and capsule. It is similar to *C. rufipes* Seub. due to its white flowers and rusty hairs on the leaf-sheaths, but it can be readily distinguished from the latter by its connate spathe base (*vs.* free base); auriculate medial petal without medial constriction (*vs.* entire medial petal with medial constriction); light-brown, ellipsoid, dehiscent capsules (*vs.* pearly-white to silvery, globose, crustaceous capsules); and by its free, ornamented seeds (*vs.* seeds fused to the capsule septa, forming a dispersal unit, with smooth testa). The gross floral morphology of *C. rufipes* is much more similar to the *C. benghalensis* than the one of *C. huntii*, possessing only the white petals in common.

*Commelina huntii* is most similar to *C. obliqua* Vahl due to its oblique leaf blades, connate spathe base, dehiscent capsules, and ventral seeds free with foveolate testa. Despite this, *C. huntii* can be distinguished from *C. obliqua* by its densely setose leaf-sheath margins with rusty to rusty brown hairs (*vs.* leaf-sheath margins long-ciliate with light to medium to dark brown to atro-vinaceous hairs); petals white (*vs.* blue to light blue to lilac to pale lilac), paired petals broadly rhomboid to rhomboid reniform (*vs.* broadly ovate to broadly ovate reniform), medial petal cucullate and biauriculate (*vs.* involute and entire); anthers of the lateral stamens light-yellow to cream with margins tinted vinaceous (*vs.* completely orange); ovary and capsules sparsely black papillate (*vs.* smooth); 1–2 capsules per spathe (*vs.* 5–7); seeds with peach-colored farina (*vs.* seeds white farinose), and dorsal locule seeds with shallowly foveolate testa (*vs.* rugose foveolate testa).

**Figure 6.**
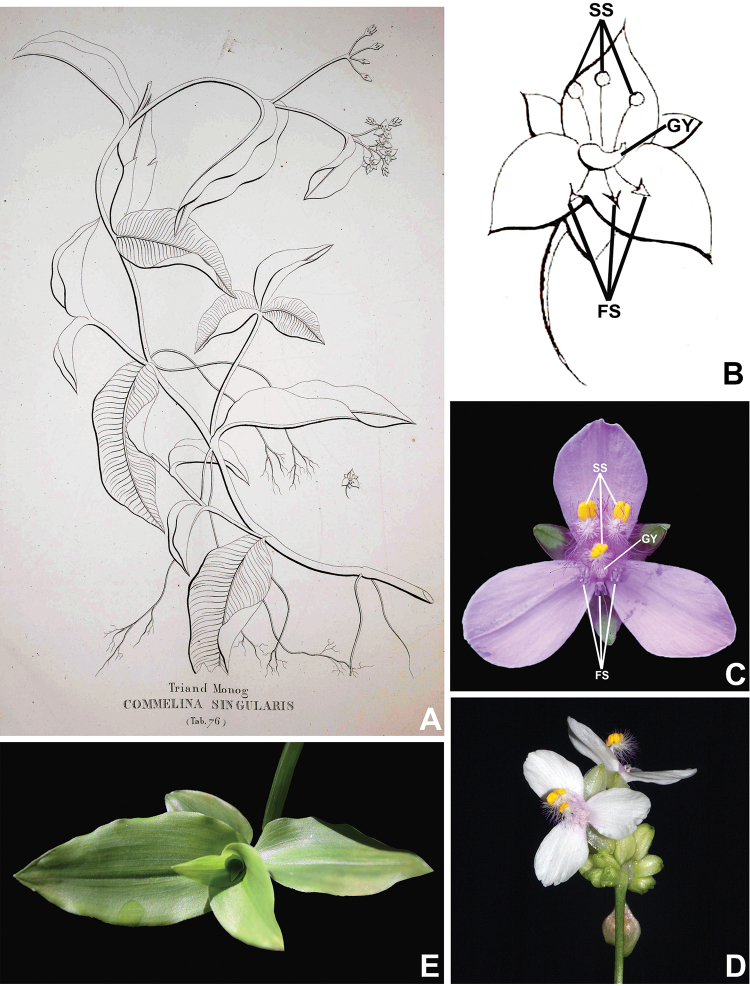
*Commelina singularis* Vell. **A–B** Original plate of Vellozo’s *C. singularis*: **A** line drawings of habit, inflorescence and floral characters **B** detail of floral characters. **C**, photos of a natural population of *Tripogandra diuretica* from the Jardim Botânico Rio de Janeiro, RJ: detail of the inflorescence, showing flowers with white corolla **D–E** photos of *T. diuretica* from the municipality of Petrópolis, RJ: **D** detail of floral characters of a flower with lilac corolla **E** detail showing the leaves (note the parallel venation characteristic of the species) **SS** sterile stamens; **FS** fertile stamens; **Gy** gynoecium. Photo of *C. singularis* plate from Biodiversity Heritage Library; All field photos by M.O.O. Pellegrini.

#### 5. Commelina obliqua

Taxon classificationPlantaeCommelinalesCommelinaceae

Vahl, Enum. Pl. 2: 172. 1806.

Fig. 1E–G

##### Lectotype

(designated by Hunt 1994). *s.loc.*, cultivated in France, ex horto Celsii, Ventenat s.n. (C barcode C10009563!).

##### Selected specimens seen.

BRAZIL. Rio de Janeiro: Guapimirim, trilha das Andorinhas, 20 Dec 1995, J.A. Lira Neto 145 (RB). Itatiaia, Parque Nacional do Itatiaia, parte baixa, atrás da casa do pesquisador, 21 Jan 2012, M.O.O. Pellegrini & L.S. Sylvestre 188 (RB). Nova Friburgo, Macaé de Cima, Reserva Ecológica Municipal de Macaé de Cima, estrada de terra próximo ao Hotel São João, 19 Jan 1999, J. Anderson et al. 9912 (UEC). Nova Iguaçu, Serra do Tinguá, Reserva Biológica do Tinguá, 13 May 1943, Guerra s.n. (NY 498159). Paraty, Fazenda São Lourenço, 17 Nov 1993, E. Martins s.n. (UEC 29410). Petrópolis, Quitandinha, Pedra do Quitandinha, 2 May 2010, M.O.O. Pellegrini 2 (RFA). Resende, margem da rodovia Dutra, Km 302 sentido RJ, ao lado da Light, próximo ao Rio Paraíba do Sul, 9 Jun 2012, M.O.O. Pellegrini et al. 231 (RB). Silva Jardim, 31 Jan 2015, L.S.B. Calazans 485 (RB). Teresópolis, Serra dos Órgãos, 26 Oct 1949, E. Pereira 635 (RB, US).

##### Distribution and habitat.

Mexico to Argentina being very common in shady disturbed areas such as road sides, gardens and forest margins, and in agricultural fields. It is less commonly found growing in drier regions and rocky outcrops (Fig. 2).

##### Phenology.

Throughout the year, but especially in the rainy season.

##### Conservation status.

Following the IUCN recommendations (IUCN 2001), as currently circumscribed, *C. obliqua* should be considered as Least Concern (LC) in the state of Rio de Janeiro and worldwide.

##### Morphological notes.

A great deal of morphological variation can be observed in *C. obliqua* and in its current circumscription. It comprises plants from small to large stature (sometimes more than a 1.5 m high); stems from creeping with ascending apex to erect to sub-scandent, and thin and fibrous to robust and somewhat succulent stems. The leaves can vary from 4–20 cm long, from glabrous to scabrous to pilose, and from green to dark green to vinaceous abaxially. Flower size and color also vary, which as in *C. erecta* seems to be environmentally influenced, probably by differences in soil pH and light intensity (Pellegrini, pers. obs.). The petals of *C. obliqua* can range from intense blue to light blue, sometimes varying from lilac to pale lilac. *Commelina obliqua* likely represents a species complex and biosystematic studies are necessary in order to better understand and elucidate its boundaries. Until this is addressed we believe that a wide circumscription, as presented here, is currently the best way to deal with this taxon.

#### 6. Commelina rufipes

Taxon classificationPlantaeCommelinalesCommelinaceae

Seub., in Martius Fl. Bras. 3(1): 265. 1855.

Figs 1H–J

##### Distribution and habitat.

Mexico to Southeastern Brazil, being found in the understory of preserved rainforests, in the Amazon and Atlantic domains, as well as in gallery forests in the Cerrado biome. It is a rare species in the Atlantic Forest and Cerrado biomes, with most of its collections being from the Amazon Forest.

##### Conservation status.

As abovementioned, *C. rufipes* is locally rare in the state of Rio de Janeiro and not as frequent in the field as the blue flowered species of the genus. Despite its wide distribution, it seems to occur only in preserved rainforests, forming dense but isolated populations. Data regarding its reproductive cycle would be of great value for this species’ conservation. Following the IUCN recommendations (IUCN 2001), *C. rufipes* as currently circumscribed should be considered as Least Concern (LC) in its worldwide distribution.

##### Taxonomical notes.

We currently accept two varieties within this species (sensu Faden & Hunt 1987). The floral morphology of both varieties of *C. rufipes* is poorly understood as little reproductive material exists. However there seems to be no morphological overlap in vegetative characters and very little overlap in their distributions. Further biosystematic study, focusing especially on floral morphology, would be most useful in evaluating their boundaries and taxonomic status.

#### 6a. Commelina rufipes var. glabrata

Taxon classificationPlantaeCommelinalesCommelinaceae

(D.R.Hunt) Faden & D.R.Hunt, Ann. Missouri Bot. Gard. 74(1): 122. 1987.

Fig. 1H

Commelinopsis glabrata D.R.Hunt, (1981: 195). Holotype. TRINIDAD. Irois Forest district, 25 January 1928, Broadway 6716 (K barcode K 000363259!).

##### Specimens seen.

BRAZIL. Rio de Janeiro: Cardoso Moreira, Fazenda Santa Rosa, 11 Jan 2014, I.G. Costa 319 (RB). Santa Maria Madalena, Parque Estadual do Desengano, Serra da Agulha, Fazenda Agulha do Imbé, between Santa Maria Madalena and Santo Antônio do Imbé, 23 February 1983, T.C. Plowman & H.C. Lima 12933 (US).

##### Taxonomical notes.

Few collections of this variety are known for the Southeastern region of Brazil, with several specimens previously identified as C. rufipes var. glabrata actually representing the herein described *C. huntii*.

#### 6b. Commelina rufipes Seub. var. rufipes

Taxon classificationPlantaeCommelinalesCommelinaceae

, in Martius Fl. Bras. 3(1): 265. 1855.

Fig. 1I–J

##### Lectotype

(designated here). BRAZIL. São Paulo: s.loc., 1817, C.F.P. Martius 76 (M barcode M0210921!). Epitype (designated here). BRAZIL. São Paulo: Bertioga, estrada Bertioga/São Sebastião, bairro São Rafael, 25 Oct 2007, R.C. Forzza et al. 4823 (RB barcode RB00515585!)

##### Selected specimens seen.

BRAZIL. Rio de Janeiro: Duque de Caxias, Reserva da Petrobrás, trilha para a barragem, 28 August 1997, J.A. Lira Neto et al. 696 (RB). Magé, 1 November 1983, R.R. Guedes et al. 537 (RB). Sapucaia, estrada que liga Sapucaia das Terras Frias até o Rio Vermelho, 13 March 1981, M.G.A. Lobo 223 (RB). Silva Jardim, Reserva Biológica de Poço das Antas, Juturnaíba, trilha Rodolfo Norte, caminho para a Pelonha, 18 August 1995, J.M.A. Braga et al. 2735 (RB). Teresópolis, Parque Nacional da Serra dos Órgãos, trilha para a Pedra do Sino, da entrada até a primeira cachoeira, 14 Jul 2011, J.A. Lombardi 8616 (HRCB, UPCB)

##### Nomenclatural notes.

When describing *C. rufipes*, Seubert (1855) only mentions that his new species was based on a Martius specimen, at M. After searching the M collection, we found just two specimens from this collector — *Martius 76* (M0210921) and *Martius 77* (M0210920). Since the specimen *Martius 76* was clearly annotated in Seubert’s handwriting it is the obvious choice for a lectotype. Nonetheless, Seubert’s original description makes it clear that all available specimens had few if any flowers, which matches the specimens found by us at M. This has caused great taxonomic problems over the years, with this name being ascribed to a number of different genera (*i.e. Athyrocarpus* Schltdl., *Commelina*, *Commelinopsis* Pichon, and *Phaeosphaerion* Hassk.), and as either accepted or as a synonym by different authors (Faden & Hunt 1987). Thus, in accordance to the *Code* (McNeill et al., 2012, Art. 9.8), we also designate a well-preserved flowering specimen as an epitype, to avoid further taxonomic and nomenclatural problems.

##### Taxonomical notes.

Apart from the obvious difference in indumenta, the leaves of C. rufipes var. rufipes tend to be thinner (lanceolate to elliptic-lanceolate), with a cuneate base and acute apex, while the leaves of C. rufipes var. glabrata tend to be wider (ovate-elliptic to ovate), with a round to obtuse base and acuminate apex.

### Excluded name

After a thorough analysis of Vellozo’s description and original illustration for *C. singularis* Vell., it became clear that this species does not belong in the genus *Commelina*. This name is better placed under the synonymy of *Tripogandra diuretica* (Mart.) Handlos (**Syn. nov.**), and the necessary taxonomic and nomenclatural comments and typifications are made below.

#### Commelina singularis

Taxon classificationPlantaeCommelinalesCommelinaceae

Vell., Fl. Flumin.: 31. 1829.

Fig. 6

##### Lectotype

(designated here). [illustration] Original parchment plate of Flora fluminensis in the Manuscript Section of the Biblioteca Nacional do Rio de Janeiro [mss1095062_080] and later published in Vellozo, Fl. flumin. Icon. 1: t. 76, pro parte, flowers and inflorescence only. Epitype (designated here). BRAZIL. Rio de Janeiro: Rio de Janeiro, Área do Jardim Botânico do Rio de Janeiro, fl., fr., 21 Dec 1995, J.A. Lira Neto 194 (RB 2ex, barcode RB00685321!).

##### Accepted name.

*Tripogandra diuretica* (Mart.) Handlos.

##### Taxonomical notes.

Vellozo’s plate for *C. singularis* (1831: t. 76) shows a creeping plant with eudicot-like leaves (net-veined and a single apparently trifoliate leaf), not identifiable as any known species of Commelinaceae. Nevertheless, the inflorescence type (Fig. 6A, C), details of the androecium (Fig. 6B, D), and the morphological description of six dimorphic stamens, three of which are staminodial — “*Stamina sex, quorum tria nectaria mentiuntur*” — (Vellozo 1829), consistently allows this name to be associated to the genus *Tripogandra* Raf. Another remarkable feature of Vellozo’s plate is the gynoecium, which is illustrated with a very short and slightly curved style (Fig. 6B). This feature distances *C. singulars* from the genus *Commelina* where the style is long and sigmoid, bringing it closer to *Tripogandra*. The leaves illustrated by Vellozo belong to the genus *Polygonum* L. (Polygonaceae), which usually possesses white to pink to lilac flowers, and occurs in the same marshes as *T. diuretica*. This confusion is apparently common in Brazilian herbaria, where *Polygonum* specimens are commonly misidentified as commelinaceous taxa (Pellegrini pers. obs.).

Vellozo (1829: 31) also mentions that *C. singularis* is found growing in slow-water environments — “*Aquis stagnantibus, et confluentibus habitat*” —. Only *T. diuretica* and *T. warmingiana* (Seub.) Handlos occur in the state of Rio de Janeiro; the first being very common, extremely variable in size and flower morphology, and normally occurring in marshy areas; the second being very rare, uniformly small in size and flower morphology, and occurring in drier areas (Pellegrini et al. 2013). Thus, *C. singularis* is here regarded as a synonym of *T. diuretica*. In accordance to the *Code* (McNeill et al., 2012, Art. 9.8), in order to avoid future confusions and to fix the application of this name, we herein designate an epitype for *C. singularis*.

## Discussion

Our work has reaffirmed the importance of thorough descriptions, fieldwork, photographs, spirit collections, and cultivation of specimens to better understand the taxonomy and systematics of intricate genera such as *Commelina*. This genus in particular poses problems as floral characters are difficult to observe in dried specimens (*e.g.* Faden 1993, 2008, 2012; Nampy et al. 2013), which calls for particular attention to be paid to adequate description of these in any new species (Faden 2008). Historically there are examples where either floral, fruit, or seed characters are only incompletely described, or even omitted. In some cases, the available specimens possess such strikingly different habit or vegetative characters, that the name can be easily applied (*e.g.* Faden et al. 2009). Nevertheless, in most cases, the lack of appropriately detailed description may cause confusion or prevent identification and the application of a correct name. Capturing the range of a species’ phenotype is also important and population studies have shown to be of great use, especially in the Neotropical species, allowing us to record and compare wide ranges of morphology. The description of new taxa, based on few and odd specimens needs to be carefully considered, and is a strategy that tends to inflate the description of unnecessary or invalid new species and infraspecific taxa.

Characters such as inflorescence position and morphology, spathe shape and conation, petal and fruit morphology, and seed ornamentation play important roles in species distinction and delimitation, in *Commelina*. Nevertheless, characters once thought to be useful in species delimitation such as indumenta and leaf shape have shown to be highly variable within the same species and thus not completely reliable. This is easily observed in all Neotropical species, and most of the wide-spread species (*e.g. C. benghalensis*, *C. diffusa*, and *C. erecta*). Growth-form and subterraneous system morphology are also potentially interesting for the taxonomy of *Commelina* worldwide. On the other hand, most of the morphological characters pointed out by previous authors (*e.g.* Faden 1993, 2008; Nampy et al. 2013) as key to the taxonomy of the genus are mostly difficult to observe in herbaria specimens. Thus, work to expand and refine the morphological tools available to workers in this group should be ongoing. It is also apparent that some species still need further systematic study in order to clarify their boundaries. The Pantropical *C. diffusa* complex is poorly understood in the Neotropical region and is probable that more than one species, being treated under the widely polymorphic C. diffusa subsp. diffusa. The *C. obliqua* and *C. rufipes* complexes also need critical attention. The *C. rufipes* complex seems to be exclusively Neotropical, while the *C. obliqua* complex is here confirmed to be Pantropical, reaching Asia. These two species groups are historically problematic, and many names have been accommodated under one concept or another, depending on the author. It is very likely that both complexes will need to be studied concomitantly in order to fully understand their phylogenetic history, taxonomy and nomenclature.

## Supplementary Material

XML Treatment for Commelina benghalensis

XML Treatment for Commelina diffusa

XML Treatment for Commelina erecta

XML Treatment for Commelina huntii

XML Treatment for Commelina obliqua

XML Treatment for Commelina rufipes

XML Treatment for Commelina rufipes var. glabrata

XML Treatment for Commelina rufipes Seub. var. rufipes

XML Treatment for Commelina singularis

## References

[B1] Bachman S Moat J Hill AW Torre J Scott B (2011) Supporting Red List threat assessments with GeoCAT: geospatial conservation assessment tool. In: Smith V Penev L (Eds) e-Infrastructures for data publishing in biodiversity science. ZooKeys 150: 117–126. https://doi.org/10.3897/zookeys.150.2109 10.3897/zookeys.150.2109 PMC3234434 22207809

[B2] Barreto RC (1997) Levantamento das espécies de Commelinaceae R.Br. nativas do Brasil. PhD Thesis, Universidade de São Paulo, Instituto de Biociências, São Paulo.

[B3] BFG [The Brazilian Flora Group] (2015) Growing knowledge: an overview of Seed Plant diversity in Brazil. Rodriguésia 66(4): 1085–1113. https://doi.org/10.1590/2175-7860201566411

[B4] Clarke CB (1881) Commelinaceae. In: De Candolle A (Ed.) Monographiae Phanerogamarum, (Vol. 3). Sumptibus G. Masson, Paris, 113–324.

[B5] eMonocot (2010) eMonocot. http://e-monocot.org [Version 1.0.2; accessed: 12.8.2016]

[B6] Faden RB (1991) The morphology and taxonomy of *Aneilema* R.Brown (Commelinaceae). Smithsonian Contributions to Botany (Washington, DC) 76: 1–181. https://doi.org/10.5479/si.0081024X.76

[B7] Faden RB (1992) (1052) Proposal to conserve *Commelina benghalensis* (Commelinaceae) with a conserved type under Art. 69.3. Taxon 41(2): 341–342. https://doi.org/10.2307/1222348

[B8] Faden RB (1993) The misconstrued and rare species of *Commelina* (Commelinaceae) in the eastern United States. Annals of the Missouri Botanical Garden 80(1): 208–218. https://doi.org/10.2307/2399824

[B9] Faden RB (1998) Commelinaceae. In: Kubitzki K (Ed.) The families and genera of vascular plants (Vol. 4). Springer Verlag, Berlin, 109–128. https://doi.org/10.1007/978-3-662-03531-3_12

[B10] Faden RB (2000) Commelinaceae R.Brown: Spiderwort family. In: (Eds) Flora of North America North of Mexico Magnoliophyta: Alismatidae, Arecidae, Commelinidae (in part), and Zingiberidae, vol. 22. Oxford University Press, Oxford, New York, 170–197.

[B11] Faden RB (2008) New species of *Commelina* (Commelinaceae) from East and South-Central Africa. Novon 18(4): 469–479. https://doi.org/10.3417/2007025

[B12] Faden RB Hunt DR (1987) Reunion of *Phaeosphaerion* and *Commelinopsis* with *Commelina* (Commelinaceae). Annals of the Missouri Botanical Garden 74(1): 121–122. https://doi.org/10.2307/2399267

[B13] Faden RB Hunt DR (1991) The Classification of the Commelinaceae. Taxon 40(1): 19–31. https://doi.org/10.2307/1222918

[B14] Govaerts R Faden RB (2009) World checklist of selected plant families. The Board of Trustees of the Royal Botanical Gardens, Kew http://apps.kew.org/wcsp [accessed: 27.11.2016)

[B15] Herbert W (1826) *Commelina deficiens*: Two-petaled *Commelina*. Curtis’s Botanical Magazine 53: 2644.

[B16] Hunt DR (1981) Precursory notes on Commelinaceae for the Flora of Trinidad and Tobago – American Commelinaceae, vol. X. Kew Bulletin 36(1): 195–197. https://doi.org/10.2307/4119017

[B17] Hunt DR (1994) Commelinaceae. In: Davidse G Sousa-Sánchez M Chater AO (Eds) Flora Mesoamericana: Alismataceae a Cyperaceae, vol. 6. Universidad Nacional Autónoma de México, 157–173.

[B18] IBGE – Instituto Brasileiro de Geografia e Estatística (2012) Manual Técnico da vegetação Brasileira: sistema fitogeográfico, inventário das formações florestais e campestres, técnicas e manejo de coleções botânicas, procedimentos para mapeamentos, ed. 2, vol. 1. IBGE, Rio de Janeiro, 272 pp.

[B19] IUCN (2001) The IUCN red list of threatened species, version 2010.4. IUCN Red List Unit, Cambridge. http://www.iucnredlist.org [accessed: 2.6.2016]

[B20] Joseph SM Nampy S (2012) Capsule and seed morphology of *Commelina* L. (Commelinaceae) in relation to taxonomy. International Journal of Botany 8(1): 1–12. https://doi.org/10.3923/ijb.2012.1.12

[B21] McNeill J Barrie FR Buck WR Demoulin V Greuter W Hawksworth DL Herendeen PS Knapp S Marhold K Prado J Prud’Homme Van Reine WF Smith GF Wiersema JH Turland NJ (Eds) (2012) International Code of Botanical Nomenclature (Melbourne Code). Adopted by the Eighteenth International Botanical Congress Melbourne, Australia, July 2011. Regnum Vegetabile 154. A.R.G. Gantner Verlag KG, 1–240.

[B22] Nampy S Joseph SM Manudev KM (2013) The genus *Commelina* (Commelinaceae) in Andaman & Nicobar Islands, India with one new species and three new records. Phytotaxa 87(2): 19–29. https://doi.org/10.11646/phytotaxa.87.2.1

[B23] Panigo E Ramos J Lucero L Perreta M Vegetti A (2011) The inflorescence in Commelinaceae. Flora 206(4): 294–299. https://doi.org/10.1016/j.flora.2010.07.003

[B24] Pellegrini MOO (2015) Notes on the Pontederiaceae names described in Vellozo’s *Flora fluminensis*. Rodriguésia 66(3): 913–916. https://doi.org/10.1590/2175-7860201566318

[B25] Pellegrini MOO Aona-Pinheiro LYS Forzza RC (2013) Taxonomy and conservation status of *Tripogandra warmingiana* (Seub.) Handlos (Commelinaceae), a previously obscure taxon from Brazil. Phytotaxa 91(2): 39–49. https://doi.org/10.11646/phytotaxa.91.2.2

[B26] Pellegrini MOO Carvalho MLS (2016) The identity and application of *Coletia madida* and notes on the typification of Mayacaceae. Taxon 65(3): 605–609. https://doi.org/10.12705/653.12

[B27] Pellegrini MOO Forzza RC Sakuragui CM (2015) A nomenclatural and taxonomic review of *Tradescantia* L. (Commelinaceae) species described in Vellozo’s *Flora fluminensis* and notes on Brazilian *Tradescantia*. Taxon 64(1): 151–155. https://doi.org/10.12705/641.3

[B28] Radford AE Dickison WC Massey JR Bell CR (1974) Vascular Plant Systematics. Harper & Row Publishers, New York, 891 pp.

[B29] Seubert MA (1855) Commelinaceae. In: Martius CFP Eichler AW (Eds) Flora Brasiliensis, vol. 3, part 1. Leipzig apud Frid. Fleischer, Munich, 233–270. [t. 32–37]

[B30] Spjut RW (1994) A systematic treatment of fruit types. The New York Botanical Garden, New York, 181 pp.

[B31] Stafleu FA Cowan RS (1979) Taxonomic literature. A selective guide to botanical publications and collections with dates, commentaries and types (2^nd^ edn , Vol. 2). Regnum Vegetabile 94. ARG Gantner Verlag, Rugell, 991 pp.

[B32] The Plant List (2013) The Plant List http://www.theplantlist.org [Version 1.1; accessed: 12.8.2015]

[B33] Thiers B (continually updated) Index Herbariorum: A global directory of public herbaria and associated staff. New York Botanical Gardens’ Virtual Herbarium http://sweetgun.nybg.org/ih [accessed: 15.5.2016]

[B34] Tropicos.org (2014) Missouri Botanical Garden. http://www.tropicos.org/ [accessed: accessed: 12.8.2016]

[B35] Vellozo JMC (1825) (1829) Florae fluminensis. Typographia Nationali, Rio de Janeiro, 352 pp.

[B36] Vellozo JMC (1827) (1831) Florae fluminensis Icones (Vol. 1). Lithogr. Senefelder, Paris, 153 pp.

[B37] Weberling F (1965) Typology of inflorescences. Botanical Journal Linnean Society 59: 15–221. https://doi.org/10.1111/j.1095-8339.1965.tb00058.x

[B38] Weberling F (1989) Morphology of flowers and inflorescences. Cambridge University Press, Cambridge, 348 pp.

